# Control of electronic polarization *via* charge ordering and electron transfer: electronic ferroelectrics and electronic pyroelectrics

**DOI:** 10.1039/d3sc03432a

**Published:** 2023-09-08

**Authors:** Sheng-Qun Su, Shu-Qi Wu, Shinji Kanegawa, Kaoru Yamamoto, Osamu Sato

**Affiliations:** a Institute for Materials Chemistry and Engineering & IRCCS, Kyushu University 744 Motooka, Nishi-ku Fukuoka 819-0395 Japan sato@cm.kyushu-u.ac.jp; b Department of Applied Physics, Okayama University of Science Okayama 700-0005 Japan

## Abstract

Ferroelectric, pyroelectric, and piezoelectric compounds whose electric polarization properties can be controlled by external stimuli such as electric field, temperature, and pressure have various applications, including ferroelectric memory materials, sensors, and thermal energy-conversion devices. Numerous polarization switching compounds, particularly molecular ferroelectrics and pyroelectrics, have been developed. In these materials, the polarization switching usually proceeds *via* ion displacement and reorientation of polar molecules, which are responsible for the change in ionic polarization and orientational polarization, respectively. Recently, the development of electronic ferroelectrics, in which the mechanism of polarization change is charge ordering and electron transfer, has attracted great attention. In this article, representative examples of electronic ferroelectrics are summarized, including (TMTTF)_2_X (TMTTF = tetramethyl-tetrathiafulvalene, X = anion), *α*-(BEDT-TTF)_2_I_3_ (BEDT-TTF = bis(ethylenedithio)-tetrathiafulvalene), TTF–CA (TTF = tetrathiafulvalene, CA = *p*-chloranil), and [(*n*-C_3_H_7_)_4_N][Fe^III^Fe^II^(dto)_3_] (dto = 1,2-dithiooxalate = C_2_O_2_S_2_). Furthermore, polarization switching materials using directional electron transfer in nonferroelectrics, the so-called electronic pyroelectrics, such as [(Cr(*SS*-cth))(Co(*RR*-cth))(*μ*-dhbq)](PF_6_)_3_ (dhbq = deprotonated 2,5-dihydroxy-1,4-benzoquinone, cth = 5,5,7,12,12,14-hexamethyl-1,4,8,11-tetraaza-cyclotetradecane), are introduced. Future prospects are also discussed, particularly the development of new properties in polarization switching through the manipulation of electronic polarization in electronic ferroelectrics and electronic pyroelectrics by taking advantage of the inherent properties of electrons.

## Abstract 2

Advances in molecular electronic ferroelectrics are summarized. In addition, electronic pyroelectrics using electron transfer is introduced as a promising alternative in the development of polarization switching materials.

## Graphical abstract

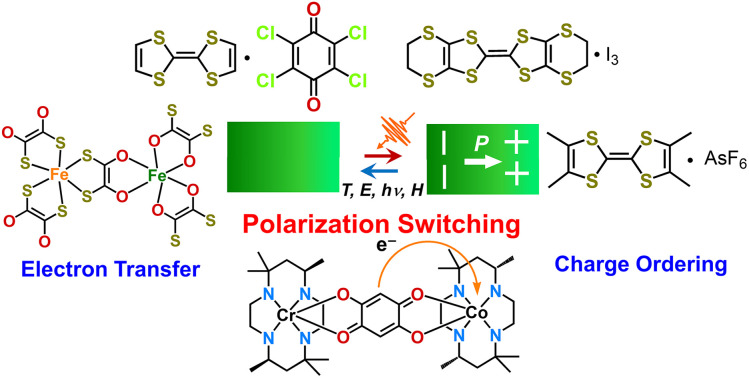

## 1. Introduction

Molecular assemblies whose physical properties can be switched in response to external stimuli have been extensively studied, leading to the development of numerous dynamic molecular crystals with switchable magnetic, electric, optical, and mechanical properties.^1–7^ The change in the physical properties of such materials results from the induction of dynamic motions of electrons, ions, and molecules, that is, electron transfer, ion displacement, and molecular reorientation, among others.^8–15^

Polarization is among the most basic and important properties of organic and inorganic compounds. Therefore, the development of polarization switching materials such as ferroelectrics, pyroelectrics, and piezoelectrics and their characterization are important subjects in materials science.^14,16–19^ Polarization changes in polar compounds are widely employed for diverse applications, including IR light detectors, pyroelectric sensors, and thermal energy-conversion devices. In particular, polarization switching induced by an electric field is the primary mechanism of ferroelectric memory devices. Polarization switching usually occurs as a result of the displacement of ions or the reorientation of polar molecules. Conversely, materials in which charge ordering and electron transfer are the source of polarization switching, the so-called electronic ferroelectrics, have recently attracted significant attention.^18,20–23^ In these materials, electrons are expected to move faster than ions and molecules, and the polarization can be modulated *via* light-induced excitation of the charge-transfer band. The polarization of compounds with electron spin can potentially be manipulated by applying a magnetic field. The durability of the switching behavior in electronic ferroelectric compounds is expected to be better than that in normal ferroelectric compounds because the ion displacement and molecular reorientation are usually accompanied by a relatively large lattice distortion.

Furthermore, more recently, the control of electronic polarization in nonferroelectrics has been reported. The electronic polarization switching is realized through a precise molecular design to ensure that electron transfer in molecules occurs in the same direction throughout the crystal. This approach to develop polarization switchable compounds is different from the typical approach that is based on the development of ferroelectrics. Meanwhile, polarization-switchable polar compounds (nonferroelectrics) *via* vectorial electron transfer are called electronic pyroelectrics.

**Fig. 1:**
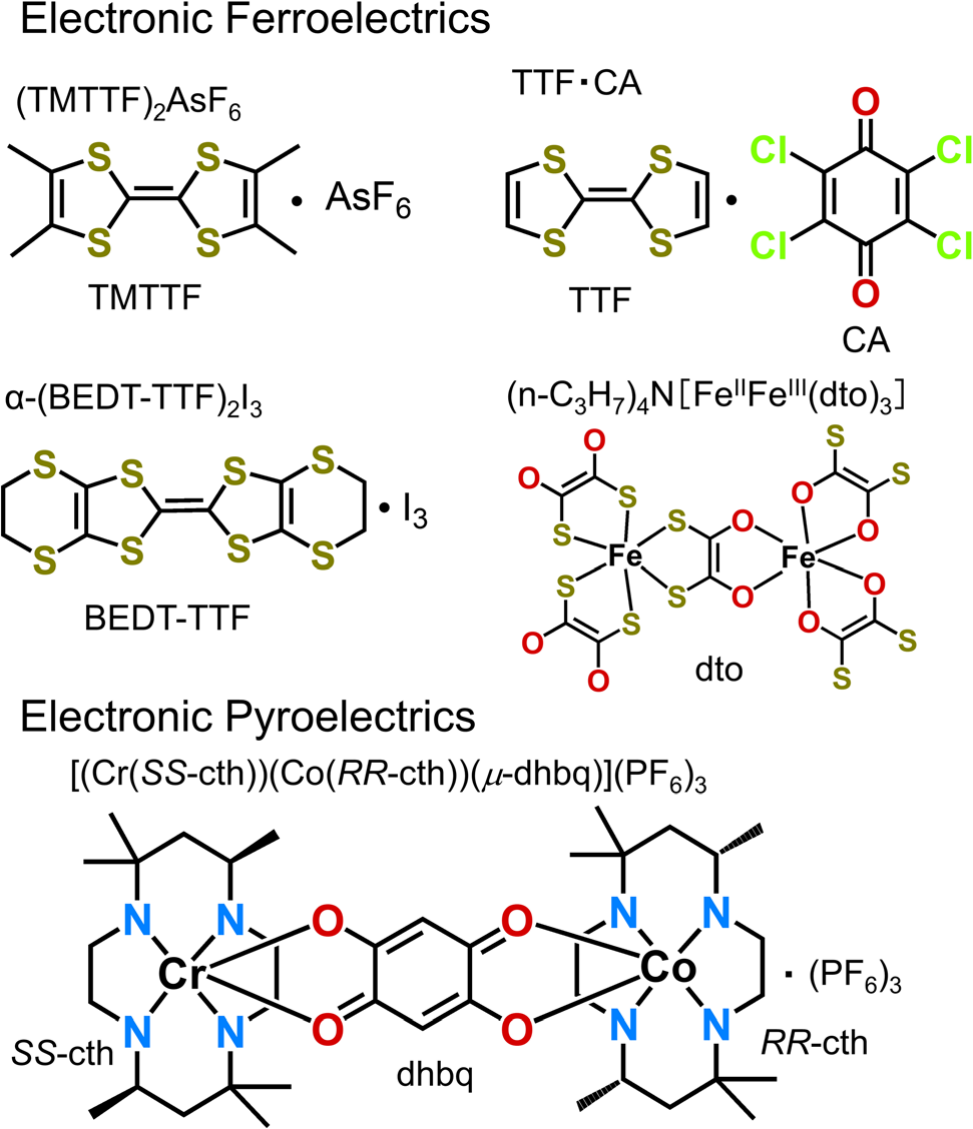
Chemical structures of representative electronic ferroelectrics and electronic pyroelectrics: (TMTTF)_2_AsF_6_, *α*-(BEDT-TTF)_2_I_3_, TTF–CA, (*n*-C_3_H_7_)_4_N[Fe^II^Fe^III^(dto)_3_], and [(Cr(*SS*-cth))(Co(*RR*-cth))(*μ*-dhbq)](PF_6_)_3_.

Considering the increasing research attention that is recently being paid to the development of electronic ferroelectrics and electronic pyroelectrics,^24,25^ we summarize in this article representative examples of molecular electronic ferroelectrics (Fig. 1), which include (TMTTF)_2_X (TMTTF = tetramethyl-tetrathiafulvalene, X = anion), *α*-(BEDT-TTF)_2_I_3_ (BEDT-TTF = bis(ethylenedithio)-tetrathiafulvalene), TTF–CA (TTF = tetrathiafulvalene, CA = *p*-chloranil), and [(*n*-C_3_H_7_)_4_N][Fe^III^Fe^II^(dto)_3_] (dto = 1,2-dithiooxalate = C_2_O_2_S_2_), and electronic pyroelectrics such as [(Cr(*SS*-cth))(Co(*RR*-cth))(*μ*-dhbq)](PF_6_)_3_ (dhbq = deprotonated 2,5-dihydroxy-1,4-benzoquinone, cth = 5,5,7,12,12,14-hexamethyl-1,4,8,11-tetraaza-cyclotetradecane).

## 2. Electric polarization

### 2.1. Polarization

**Fig. 2:**
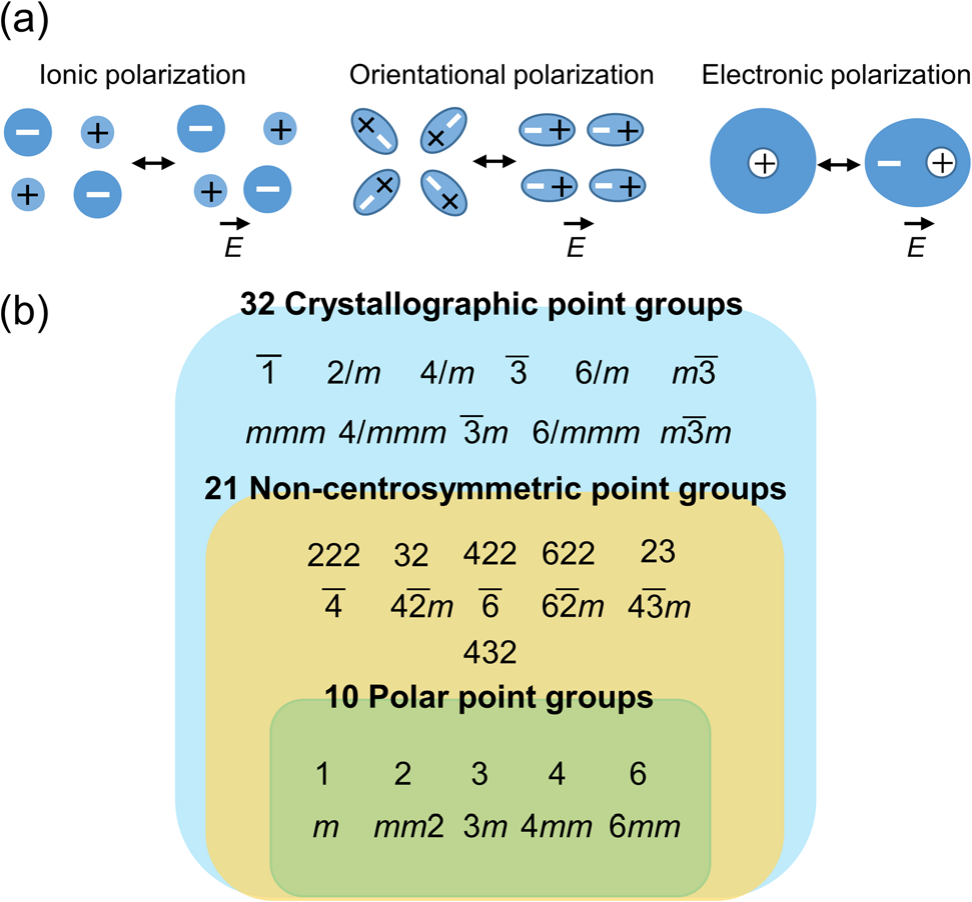
(a) Classification of electric polarization: ionic polarization, orientational polarization, and electronic polarization. (b) Crystallographic point groups. Compounds belonging to noncentrosymmetric point groups, except those classified as 432 according to the Hermann–Mauguin notation, exhibit piezoelectric properties. Compounds belonging to 10 polar point groups exhibit pyroelectric properties.

The application of an electric field (*E*) to materials induce a dipole moment (*p*), which can be expressed by the formula*p* = *αE*where *α* is the polarizability. The polarizability comprises three major components, *i.e.*, ionic polarizability (*α*_ion_), orientational polarizability (*α*_or_), and electronic polarizability (*α*_el_) (Fig. 2a). Therefore, *α* can be expressed as *α* = *α*_ion_ + *α*_or_ + *α*_el_. When an external electric field is applied to a material, ions undergo displacement along the electric field direction, which is the origin of *α*_ion_. When materials consist of polar molecules, the orientation of the molecules changes upon exposure to an electric field, resulting in *α*_or_. Meanwhile, electron clouds also undergo displacement in response to an electric field, which is the origin of *α*_el_. Notably, in some compounds, another component originating from the migration of charge carriers that form space charges, the so-called space charge polarizability (*α*_sp_), contributes to the polarization.

Compounds having polar crystal structures exhibit electric polarization in the absence of an external electric field, which is called spontaneous polarization. As shown in Fig. 2b, there are 32 crystallographic point groups,^26^ out of which 11 have a centrosymmetric structure and the remaining 21 point groups are noncentrosymmetric. Of the noncentrosymmetric 21 point groups, 10 have a polar structure with spontaneous polarization. Compounds with noncentrosymmetric point groups, except those classified as 432 according to the Hermann–Mauguin notation, have piezoelectric properties. Furthermore, polar compounds exhibit pyroelectric properties. When the polarization direction in polar compounds can be inverted by an external electric field, the compounds are ferroelectrics.

### 2.2. Polarization switching *via* electron transfer

**Fig. 3:**
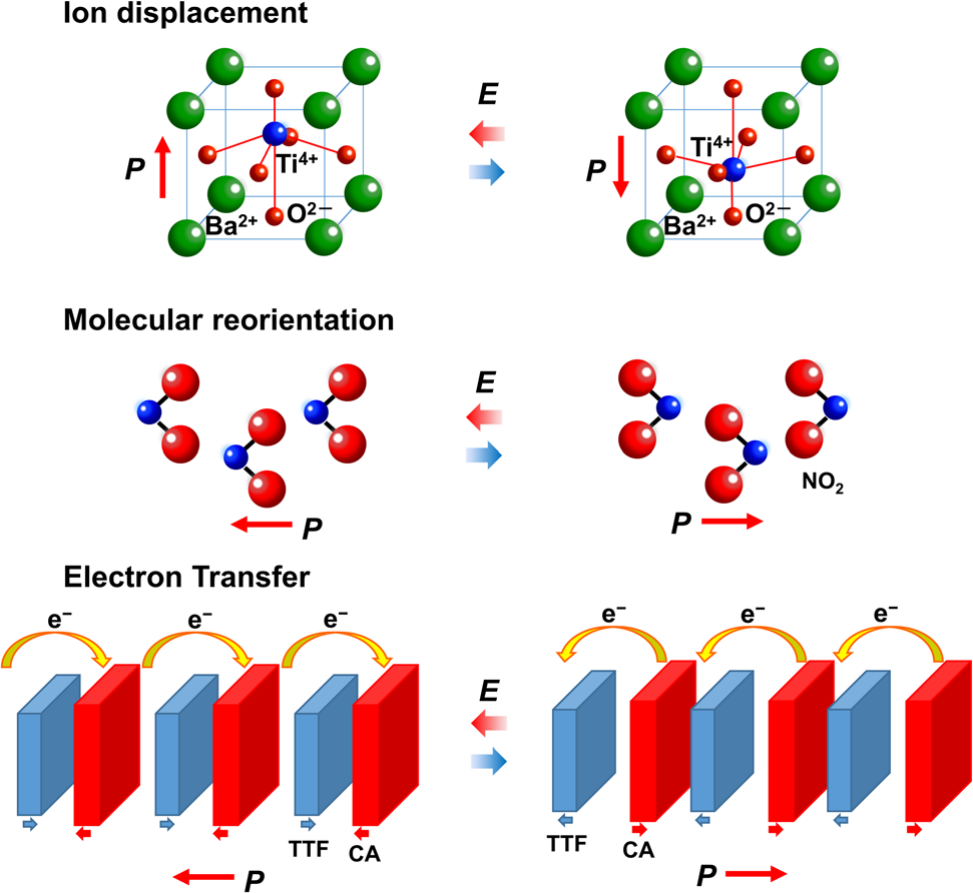
Mechanisms of polarization switching in ferroelectrics: ion displacement, molecular reorientation, and electron transfer.

As shown in Fig. 3, typical polarization switching mechanisms in ferroelectrics are ion displacement and reorientation of polar molecules. Another mechanisms are electron transfer and charge ordering (Fig. 3).^20,21,27,28^ As already described, ferroelectrics whose polarization switching mechanism is electron transfer are called electronic ferroelectrics. Several electronic ferroelectrics that exhibit a change in electronic polarization rather than in ionic and orientational polarization have been developed.

**Fig. 4:**
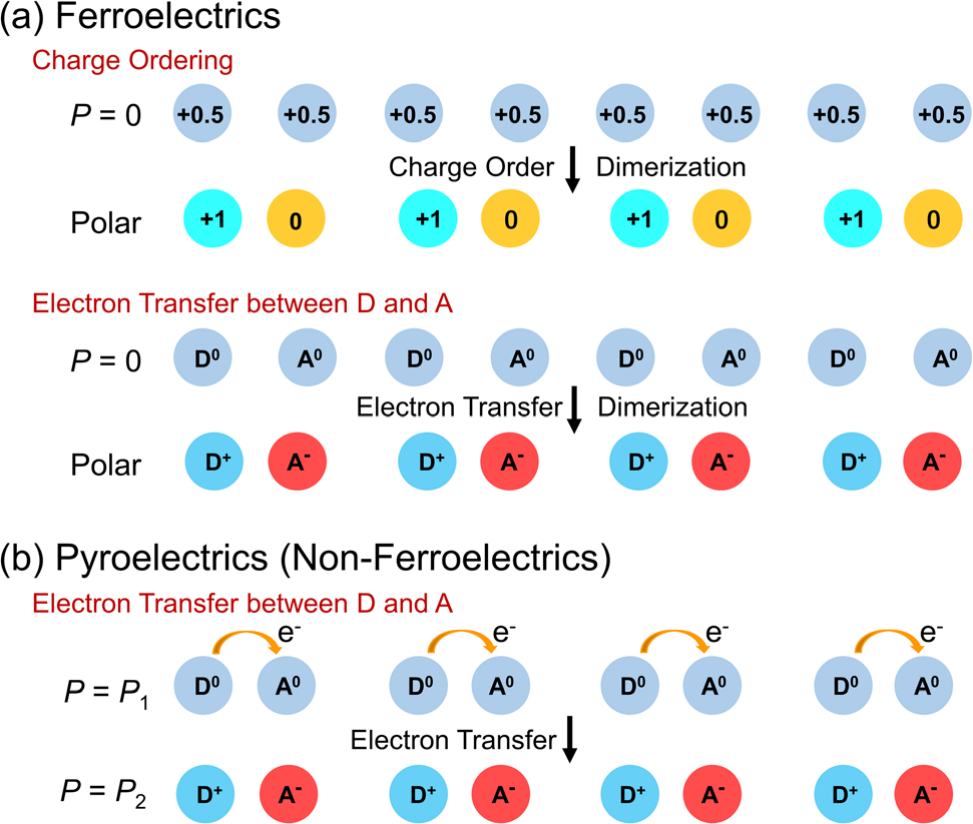
(a) Electronic polarization switching *via* charge ordering and electron transfer in ferroelectrics. (b) Electronic polarization switching *via* electron transfer in pyroelectrics.

A schematic illustration of the polarization change induced *via* charge ordering accompanied by dimerization of two molecules is shown in Fig. 4a. Molecules with charge +0.5 are aligned at the same spacing, and no macroscopic polarization is observed. In contrast, when charge ordering with dimerization is induced, macroscopic polarization, in which molecules with charge +1 and charge 0 are aligned alternately, is generated (Fig. 4a). Another example of polarization change is also shown in Fig. 4a. Again, no macroscopic polarization exists when donor molecules with charge 0 (D^0^) and acceptor molecules with charge 0 (A^0^) are aligned at the same spacing. Upon electron transfer from D^0^ to A^0^ with dimerization, macroscopic polarization is generated; in this case, molecules with charge +1 (D^+^) and charge −1 (A^−^) are aligned alternately (Fig. 4a).

Recently, nonferroelectric compounds undergoing polarization switching *via* electron transfer are being studied. Several polar compounds that exhibit switchable electronic polarization, *i.e.*, electronic pyroelectrics, have been recently developed.^25^ A schematic illustration of the polarization change in nonferroelectric polar compounds is shown in Fig. 4b. Donor (D) and acceptor (A) molecules are aligned alternately, forming pairs with a polar structure. The electron transfer between D and A induces a change in the electronic polarization between *P* = *P*_1_ and *P* = *P*_2_.

Although any polar compounds have pyroelectric properties, their pyroelectric effects are usually small. However, when the constituent molecules of a polar compound exhibit electron transfer along the same direction in the crystal, the resulting pyroelectric effects can be comparable to those of typical ferroelectrics. Furthermore, various polarization behaviors are expected to be realized in nonferroelectrics, because it is not restricted to the typical behavior of ferroelectrics, in which the low temperature phase is normally a polar phase with larger polarization value than the high temperature phase. The development of polarization-switchable compounds using electron transfer in nonferroelectrics is a promising subject in the study of dielectrics.

In the following sections, some examples of electronic ferroelectrics and electronic pyroelectrics are discussed.

## 3. Electronic ferroelectrics

### 3.1. (TMTTF)_2_X

**Fig. 5:**
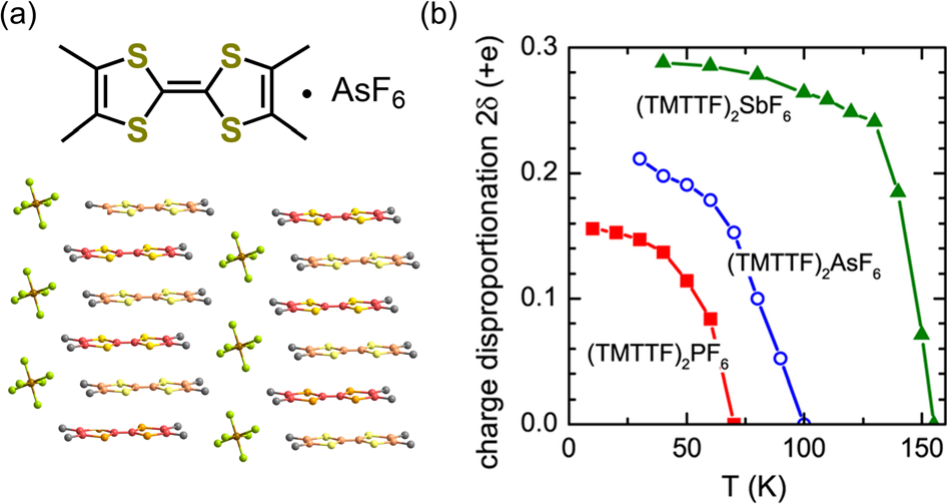
(a) Crystal structure of (TMTTF)_2_AsF_6_ (TMTTF = tetramethyl-tetrathiafulvalene).^21^ (b) Temperature dependence of the charge disproportionation (2*δ*) in (TMTTF)_2_X, where X = PF_6_, AsF_6_, and SbF_6_.^34^ Reproduced from ref. 34.

**Fig. 6:**
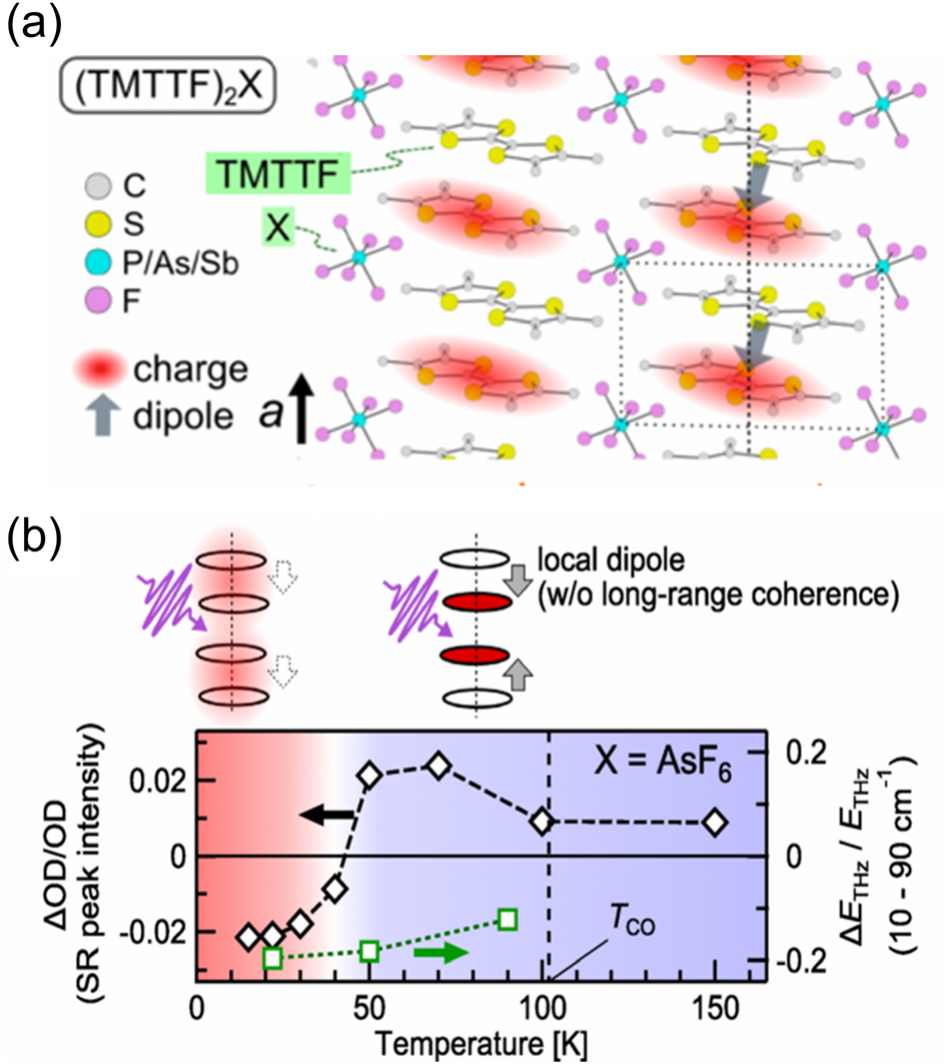
(a) Schematic of the (TMTTF)_2_X crystal with charge ordering (TMTTF = tetramethyl-tetrathiafulvalene, X = anion). Gray arrows represent the expected electric dipole.^36^ Reproduced from ref. 36. (b) Temperature dependence of the photoinduced changes in the short-range peak intensity (left axis) and THz emission (right axis). Long-range charge-order melts upon photoexcitation, whereas short-range charge fluctuations are enhanced close to the charge-order phase boundary in (TMTTF)_2_X (X = AsF_6_ and PF_6_).^36^ Reproduced from ref. 36.

The generation of polarization originating from charge disproportionation, which is also called charge ordering, has been reported in quasi-one-dimensional organic charge-transfer salts, (TMTTF)_2_X (X = anion).^29,30^ The crystal structure of (TMTTF)_2_AsF_6_ is displayed in Fig. 5a, which shows that the planar TMTTF molecules stack along the *a*-axis direction. (TMTTF)_2_X exhibits a variety of electronic and magnetic properties depending on temperature and pressure, such as spin-density-wave antiferromagnetism and metallic and superconductor features.^31^ NMR measurements revealed that (TMTTF)_2_X salts exhibit charge disproportionation^27,32^ with charge ordering temperatures of approximately 67 K, 102 K, and 157 K for X = PF_6_, AsF_6_, and SbF_6_, respectively.^33,34^ According to dielectric measurements, their dielectric constant (*ε*′) exhibits a Curie-like sharp peak of *ε*′ ≈ 10^5^–10^6^ at the charge ordering temperature,^29,30^ indicating the occurrence of a ferroelectric transition.^18,19^ The charge difference between charge-rich and charge-poor sites, *i.e.*, 2*δ* (=*ρ*_rich_ − *ρ*_poor_), of (TMTTF)_2_X can be determined using various techniques.^34,35^ For instance, by means of optical spectroscopy measurements, the 2*δ* value of (TMTTF)_2_AsF_6_ at 40 K was estimated to be about 0.2*e* (Fig. 5b),^34^ which indicates that charge-rich TMTTF^+0.6^ and charge-poor TMTTF^+0.4^ are alternately arranged along the *a*-axis direction. A schematic illustration of the (TMTTF)_2_X crystal with charge ordering and the electric dipole expected for electronic ferroelectrics is shown in Fig. 6a.^36^ Furthermore, (TMTTF)_2_SbF_6_ has been reported to exhibit antiferromagnetic ordering below 8 K,^37^ which renders it a novel multiferroic compound with electronic ferroelectricity and antiferromagnetic ordering.^38^ Recently, polarization properties in response to light were investigated in electronic ferroelectric (TMTTF)_2_X compounds (Fig. 6).^36^ THz absorption spectra, which allow detecting intradimer charge disproportionation, showed that the short-range charge correlation is enhanced by light (1.55 eV femtosecond pulses) close to the charge-order phase boundary for X = PF_6_ and AsF_6_ (Fig. 6b). It should be noted that, besides the TMTTF salts discussed above (X = PF_6_, AsF_6_, and SbF_6_), several other compounds also exhibit charge ordering behavior, which include those with X = BF_4_, and ReO_4_.^39^

### 3.2. (BEDT-TTF)_2_X

**Fig. 7:**
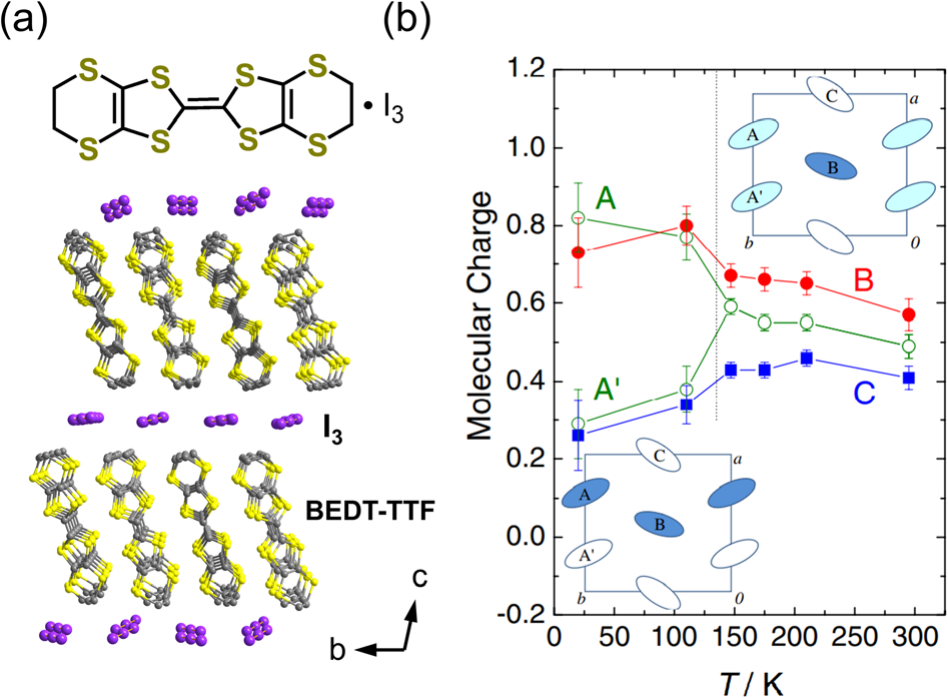
(a) Crystal structure of *α*-(BEDT-TTF)_2_I_3_ (BEDT-TTF = bis(ethylenedithio)-tetrathiafulvalene). (b) Temperature dependence of the molecular charge.^43^ Reproduced with permission from ref. 43, The Physical Society of Japan.

**Fig. 8:**
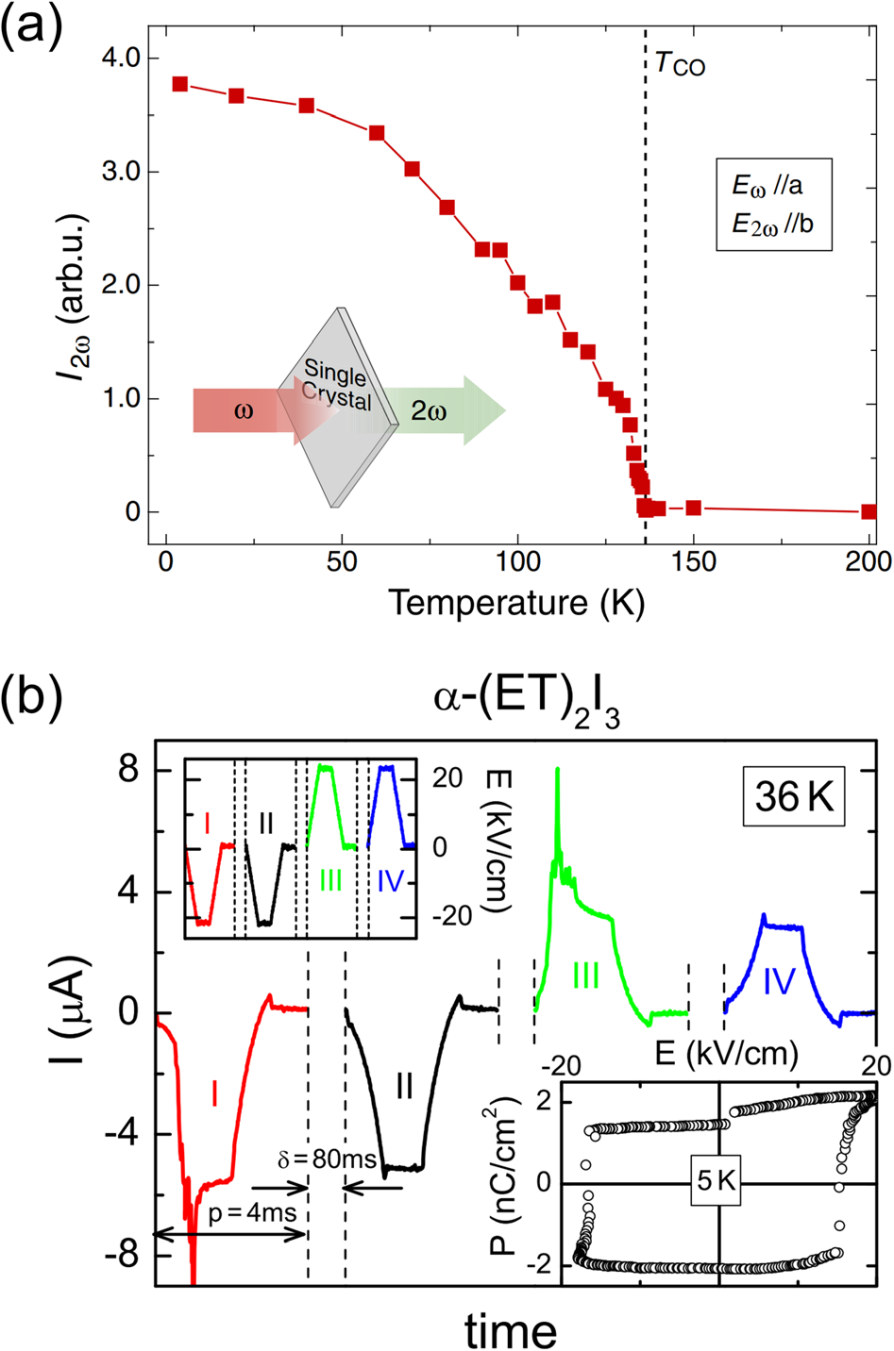
(a) Temperature dependence of the second-harmonic generation (SHG) signal.^44^ Reproduced with permission from ref. 44, The Physical Society of Japan. (b) Time-dependent current obtained from positive-up–negative-down measurements performed at 36 K. Left inset: excitation signal. Right inset: polarization (*P*) *vs.* electric field (*E*) hysteresis curve at 5 K.^46^ Reproduced with permission from ref. 46, American Physical Society.

In the two-dimensional compound *α*-(BEDT-TTF)_2_I_3_, polarization is also generated due to charge disproportionation (Fig. 7). The structure and properties of this compound have been long investigated.^40–42^ The conducting layers formed by BEDT-TTF molecules are separated by insulating anion sheets of I_3_^−^. The BEDT-TTF molecules are arranged in a herringbone structure, with the long axis of BEDT-TTF aligned along the crystallographic *c*-direction. *α*-(BEDT-TTF)_2_I_3_ exhibits charge ordering below 135 K. A single-crystal structural analysis revealed that the triclinic space group *P* at the high-temperature phase transforms to *P*_1_ at the low-temperature phase.^43^ The high-temperature phase is in the metallic state, whereas the low-temperature phase is in the insulating state.^40^*α*-(BEDT-TTF)_2_I_3_ consists of two independent alternating stacks along the *a*-axis, *i.e.*, Stack I and Stack II. In Fig. 7b, BEDT-TTF molecules in Stack I are denoted by A and A′ and those in Stack II are denoted by B and C.^44^Fig. 7b also shows the temperature dependence of the molecular charges of BEDT-TTF at A, A′, B, and C, which are estimated from the bond lengths in the molecule.^43^ A weak charge disproportionation is observed even at the high-temperature phase, and it is more pronounced below the charge ordering temperature. The charges at A, A′, B, and C are estimated to be *ρ*_A_ = 0.49(3), *ρ*_A′_ = 0.49(3), *ρ*_B_ = 0.57(4), and *ρ*_C_ = 0.41(3), respectively, at room temperature (high-temperature phase) and *ρ*_A_ = 0.82(9), *ρ*_A′_ = 0.29(9), *ρ*_B_ = 0.73(9), and *ρ*_C_ = 0.26(9), respectively, at 20 K (low-temperature phase).^43^ The temperature dependence of the second-harmonic generation (SHG) was measured upon excitation by 1400 nm light to characterize the polarization properties of the compound. As shown in Fig. 8a, an SHG signal was detected below the charge ordering temperature and its intensity increased with decreasing the temperature. This result revealed that the centrosymmetric structure at the high-temperature phase changed to a noncentrosymmetric structure at the charge ordering temperature, which is consistent with the formation of a polar structure at the low-temperature phase. Optical SHG interferometry measurements revealed that polar domain structures in the crystal varied when the crystal was annealed above the transition temperature.^45^ This behavior is indicative of the emergence of spontaneous polarization at 135 K, below which *α*-(BEDT-TTF)_2_I_3_ behaves as an electronic ferroelectric compound.^44^ The polarization properties were further investigated by performing polarization *vs.* electric field (*P*–*E*) measurements, finding that the *P*–*E* curve at 5 K showed polarization hysteresis with a saturation polarization of approximately 2 nC cm^−2^ (Fig. 8b).^46^ This small saturation polarization was suggested to result from the fact that only a fraction of polar domains were switched by the electric field because the measurement was performed far below the freezing temperature.^46^ Electronic ferroelectricity was also suggested in *κ*-(BEDT-TTF)_2_Cu[N(CN)_2_]Cl,^47^ although this is still under debate.^48^ In this compound, a THz field-induced macroscopically polarized charge-order state was observed.^49^

### 3.3. TTF–CA

**Fig. 9:**
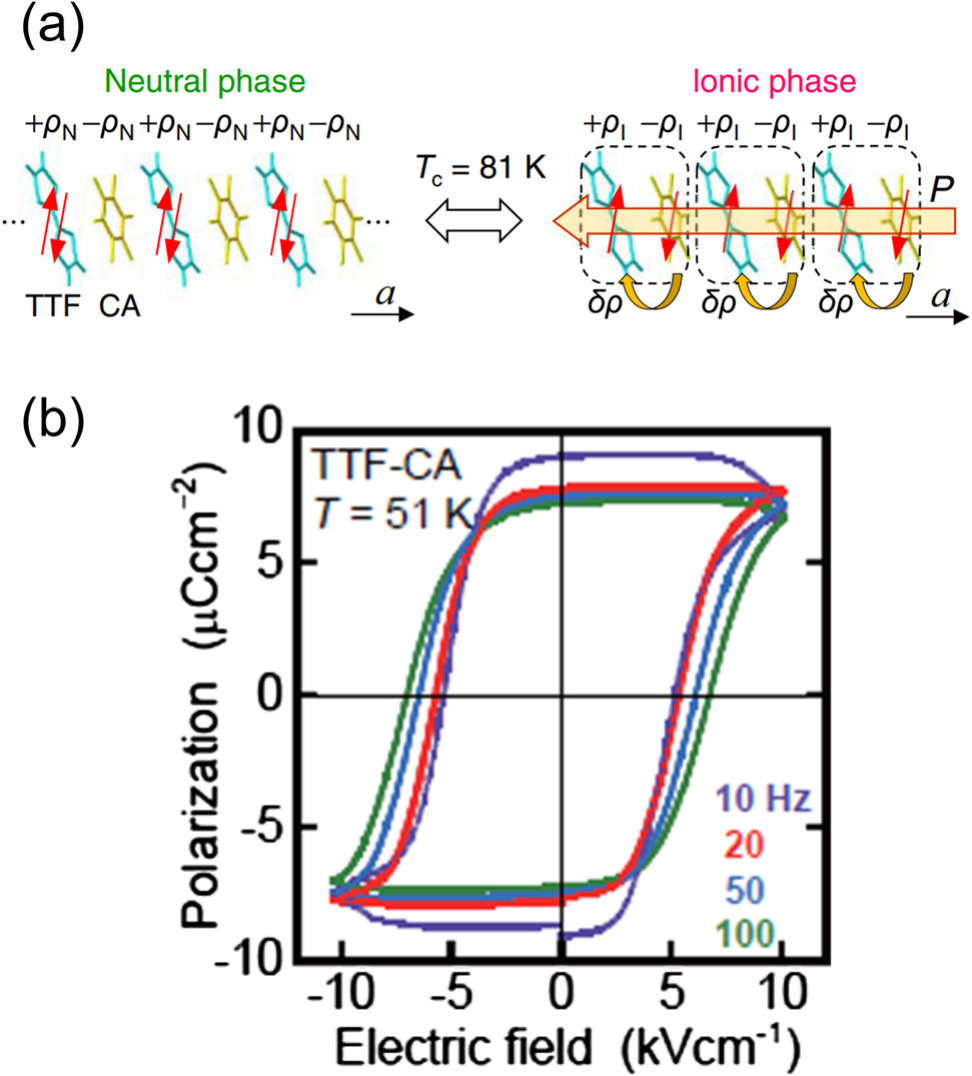
(a) Schematic illustration of the neutral-to-ionic transition in TTF–CA (TTF = tetrathiafulvalene, CA = *p*-chloranil). *P* is the ferroelectric polarization.^57^ Reproduced from ref. 57. (b) Hysteresis loops of TTF–CA with various frequencies at 51 K.^50,52^ Reproduced with permission from ref. 50, American Physical Society.

Electronic ferroelectricity was also reported in the charge-transfer complex TTF–CA,^50–52^ whose properties have been extensively investigated.^53–58^ In TTF–CA, the TTF and CA units are alternately aligned along the crystallographic *a*-direction, forming a one-dimensional structure. TTF-CA exhibits a neutral-to-ionic (N–I) transition at approximately 81 K *via* charge-transfer between TTF and CA, where the high-temperature phase is the neutral (N) phase and the low-temperature phase is the ionic (I) phase (Fig. 9a).^53^ The degree of charge-transfer (*ρ*_N_) of the N phase in TTF^+*ρ*_N_^–CA^−*ρ*_N_^ is about 0.3, whereas that (*ρ*_I_) of the I phase in TTF^+*ρ*_I_^–CA^−*ρ*_I_^ is about 0.6. The driving force of the N–I transition from N phase to I phase is an increase in the Madelung energy induced by thermal contraction upon cooling. When the gain in the Madelung energy overcomes the ionization energy of TTF–CA, the N–I transition is induced. TTF–CA exhibits ferroelectric behavior below 81 K. TTF and CA in the I phase form a donor–acceptor pair *via* displacement at the N–I transition temperature. The displacement of TTF and CA induces a polarization change (Δ*P*_ion_) according to the ion displacement mechanism. Furthermore, electron transfer between TTF and CA induces another change in polarization (Δ*P*_el_), which represents the electron transfer mechanism. Both ion displacement and electron transfer mechanisms contribute to the total polarization change (Δ*P*), which can be expressed as Δ*P* = Δ*P*_ion_ + Δ*P*_el_, where Δ*P*_el_ is greater than Δ*P*_ion_, with their sign in polarization change being opposite. Since the electron transfer mechanism dominates the polarization change, TTF–CA is an electronic ferroelectric material. As shown in Fig. 9b, the *P*–*E* curves of TTF-CA exhibit a hysteresis loop. The *P*–*E* curve at 59 K shows that the remanent polarization is 6.3 μC cm^−2^ and the coercive field is 5.4 kV cm^−1^. The spontaneous polarization was calculated to be 10.0 μC cm^−2^ according to the Berry phase approach.^59,60^ It should be noted that the analogous charge-transfer complex TTF–BA (BA = *p*-bromanil), which is ionic in the whole temperature range of study,^61^ also exhibits ferroelectric property.^62^ Spontaneous polarization is observed below 53 K. However, the ferroelectric behavior follows a typical ion displacement mechanism rather than electron transfer.

### 3.4. [(*n*-C_3_H_7_)_4_N][Fe^III^Fe^II^(dto)_3_]

**Fig. 10:**
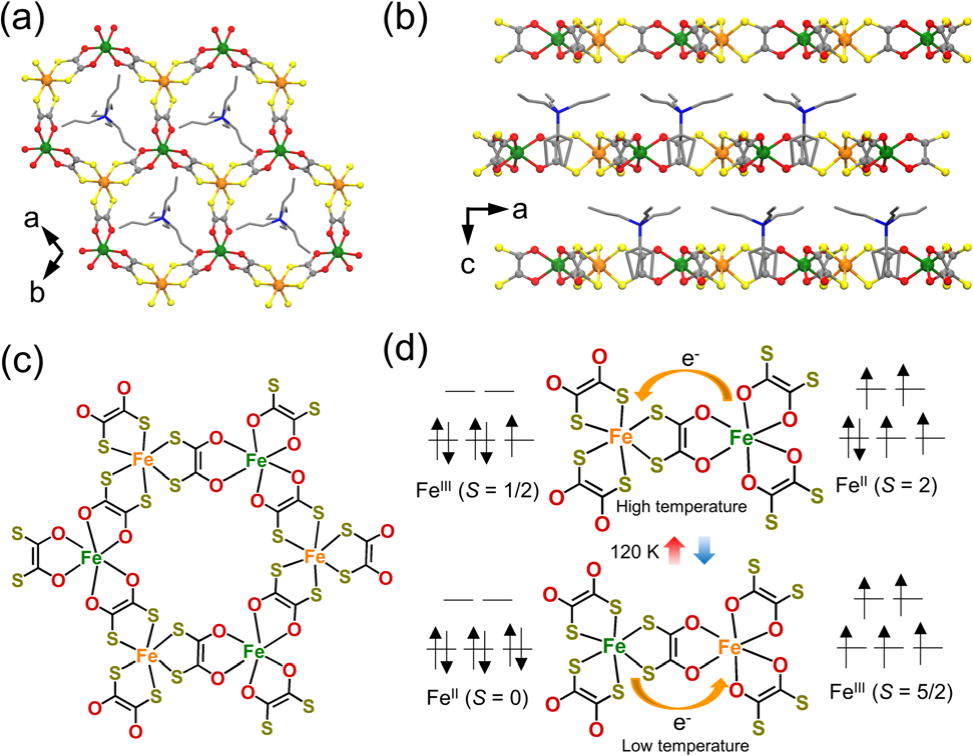
(a) Two-dimensional honeycomb network of [(*n*-C_3_H_7_)_4_N][Fe^III^Fe^II^(dto)_3_] (dto = 1,2-dithiooxalate = C_2_O_2_S_2_).^63^ (b) Projection view of the *a*–*c* plane: C, gray; O, red; S, yellow; N, blue; Fe^II^, green; Fe^III^, orange.^63^ (c) Chemical structure of the honeycomb network of (*n*-C_3_H_7_)_4_N][Fe^III^Fe^II^(dto)_3_].^64^ (d) Schematic illustration of the charge-transfer phase transition at 120 K.^64^

**Fig. 11:**
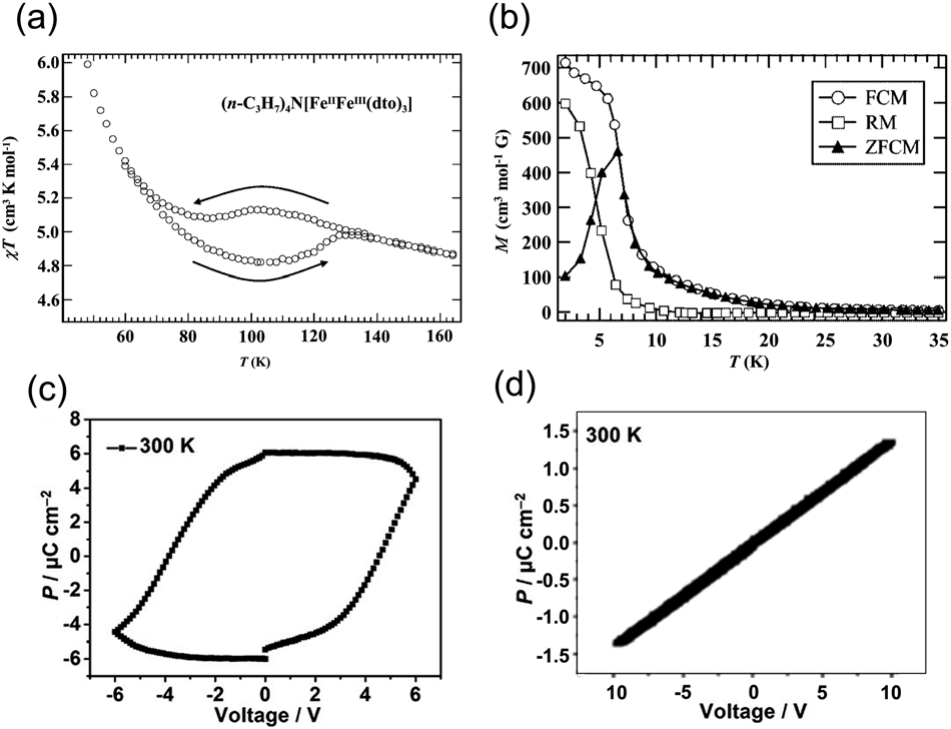
(a) Temperature dependence of the magnetic susceptibility for [(*n*-C_3_H_7_)_4_N[Fe^II^Fe^III^(dto)_3_].^64,65^ Reproduced with permission from ref. 65, Wiley. (b) Temperature dependence of the field cooled magnetization, remnant magnetization, and zero-field cooled magnetization.^64,65^ Reproduced with permission from ref. 65, Wiley. (c) Hysteresis loops of electric polarization of an [(*n*-C_3_H_7_)_4_N[Fe^II^Fe^III^(dto)_3_] film at 300 K in the in-plane direction.^63^ Reproduced with permission from ref. 63, American Chemical Society. (d) Polarization (*P*) *vs.* electric field (*E*) plot along the out-of-plane direction.^63^ Reproduced with permission from ref. 63, American Chemical Society.

Electronic ferroelectricity has also been reported in a coordination compound, [(*n*-C_3_H_7_)_4_N][Fe^III^Fe^II^(dto)_3_],^63^ whose magnetic behavior and charge-transfer phase transition behavior have been investigated.^64–68^ [(*n*-C_3_H_7_)_4_N][Fe^III^Fe^II^(dto)_3_] crystallizes in the *P*6_3_ space group (Fig. 10a and b)^64,65^ and exhibits a two-dimensional honeycomb network structure (Fig. 10a and c). One of the Fe ions, which is in the low-spin (LS) state, is coordinated by six S from the dto ligand, while another Fe ion in the high-spin (HS) state is coordinated by six O from dto. The HS Fe and LS Fe ions are alternately arranged through the dto ligand in the honeycomb structure, which is separated by an [(*n*-C_3_H_7_)_4_N]^+^ layer. The [(*n*-C_3_H_7_)_4_N][Fe^III^Fe^II^(dto)_3_] compound exhibits thermally induced electron transfer between Fe^III^ and Fe^II^ at approximately 120 K (Fig. 10d and 11a), adopting an [Fe^II^_LS_-dto-Fe^III^_HS_] structure and an [Fe^III^_LS_-dto-Fe^II^_HS_] structure at the low-temperature phase and the high-temperature phase, respectively, as follows:[Fe^II^_LS_-dto-Fe^III^_HS_] ⇄ [Fe^III^_LS_-dto-Fe^II^_HS_]

The electronic states of the Fe ions are expressed as Fe^II^_LS_ (t_2g_^6^e_g_^0^, *S* = 0) and Fe^III^_HS_ (t_2g_^3^e_g_^2^, *S* = 5/2) in the low-temperature phase and as Fe^III^_LS_ (t_2g_^5^e_g_^0^, *S* = 1/2) and Fe^II^_HS_ (t_2g_^4^e_g_^2^, *S* = 2) in the high-temperature phase. The thermal-induced electron transfer is thought to be driven by spin entropy.^69^ The spin entropy, *R* ln(2*S* + 1), in the high-temperature phase is *R* ln 10, *i.e.*, (*R* ln 2 + *R* ln 5), which is greater than that in the low-temperature phase (*R* ln 6). The contribution of the spin entropy (4.25 J K^−1^ mol^−1^) is approximately half of the total entropy gain estimated from heat capacity measurements (9.20 J K^−1^ mol^−1^). Because the energy of the entropically favorable [Fe^III^_LS_-dto-Fe^II^_HS_] state is slightly higher than that of the [Fe^II^_LS_-dto-Fe^III^_HS_] state, the transition to the [Fe^III^_LS_-dto-Fe^II^_HS_] state is realized upon heating. Furthermore, this compound exhibits ferromagnetic ordering below 10 K (Fig. 11b). Thus, [(*n*-C_3_H_7_)_4_N][Fe^III^Fe^II^(dto)_3_] has received attention owing to its molecular magnet and switchable magnetic properties. Moreover, the compounds [(*n*-C_*n*_H_2*n*+1_)_4_N][Fe^III^Fe^II^(dto)_3_] (*n* = 4, 5, 6) have also been synthesized. Among them, only [(*n*-C_4_H_9_)_4_N][Fe^III^Fe^II^(dto)_3_] exhibits charge transfer phase transition, whereas the other two compounds with *n* = 5 and 6 don't show such phase transitions.^70^

Recently, the electric polarization properties of [(*n*-C_3_H_7_)_4_N][Fe^III^Fe^II^(dto)_3_] have been investigated in depth. The *P*–*E* curve of a film of [(*n*-C_3_H_7_)_4_N][Fe^III^Fe^II^(dto)_3_] measured at 300 K shows a clear polarization hysteresis in the in-plane direction (Fig. 11c), whereas polarization hysteresis is not observed in the out-of-plane direction (Fig. 11d). Polarization hysteresis is also observed at 50 K, 120 K, 150 K, and 200 K. The saturation polarization at 300 K and 50 K in the in-plane direction was determined to be approximately 6 and 10.0 μC cm^−2^, respectively.^63^ The in-plane ferroelectric polarization calculated using the point electric charge model is ∼11.7 μC cm^−2^, which is consistent with the experimental results. The ferroelectric polarization is most likely induced by electron hopping between the Fe^II^ and Fe^III^ sites. In fact, the analogous compound [(*n*-C_3_H_7_)_4_N][Fe^III^Zn^II^(dto)_3_], in which electron hopping between Fe^III^ and Zn^II^ is prohibited, exhibits no ferroelectric signals in the in-plane direction, supporting the electron hopping mechanism in [(*n*-C_3_H_7_)_4_N][Fe^III^Fe^II^(dto)_3_].

## 4. Electronic pyroelectrics

**Fig. 12:**
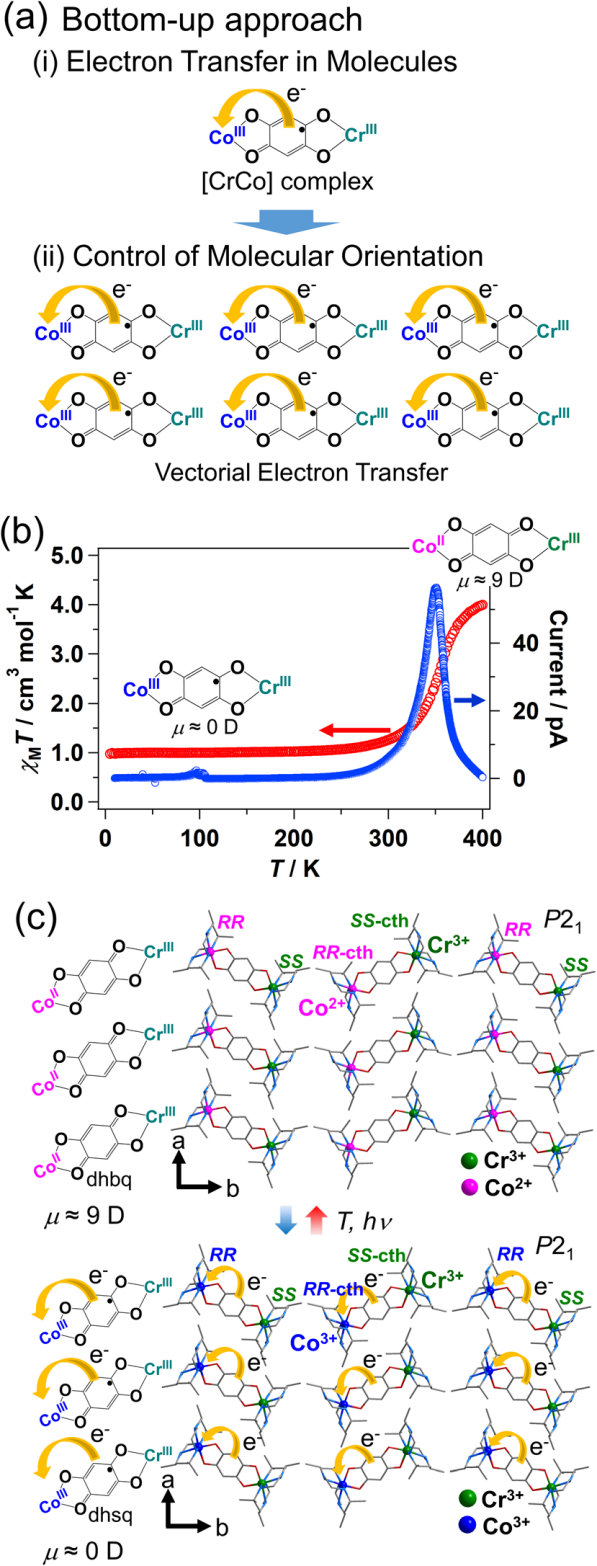
(a) Bottom-up approach to develop polarization switching compounds using electron transfer: the strategy is the combination of (i) synthesis of molecules that exhibit electron transfer (here is shown a [CrCo] dinuclear valence tautomeric complex), and (ii) control of the molecular orientation so that electron transfer occurs in the same direction in the crystal.^87^ (b) Temperature dependence of the magnetic susceptibility and pyroelectric current for [(Cr(*SS*-cth))(Co(*RR*-cth))(*μ*-dhbq)](PF_6_)_3_.^87,91^ (c) Schematic illustration of electronic polarization change *via* electron transfer between Co and the ligand.^87,92^

Polarization switching compounds can be developed using the strategy shown in Fig. 12a; the strategy is the combination of synthesis of molecules that exhibit electron transfer, and control of the molecular orientation so that electron transfer occurs in the same direction in the crystal.

### 4.1. [(Cr(*SS*-cth))(Co(*RR*-cth))(*μ*-dhbq)](PF_6_)_3_

Various compounds that exhibit electron transfer in response to external stimuli have been reported, including valence tautomeric compounds and cyanide-bridged metal complexes.^6,10,71–84^ Electron transfer in molecules is accompanied by a change in the dipole moment. However, most compounds have a centrosymmetric structure and, therefore, the change in the dipole moment induced *via* electron transfer is usually canceled out in the crystal. An example is [{Co(tpa)}_2_(*μ*-dhbq)](PF_6_)_3_·2H_2_O {tpa = tris(2-pyridylmethyl)amine and dhbq = deprotonated 2,5-dihydroxy-1,4-benzoquinone}, which exhibits electron transfer between Co and dhbq.^85^ The electron transfer process can be expressed as [Co^3+^_LS_-dhsq^3−^-Co^3+^_LS_] ⇄ [Co^2+^_HS_-dhbq^2−^-Co^3+^_LS_] (dhsq = deprotonated dihydroxysemiquinone),^85,86^ meaning that the dipole moment changes at a molecular level *via* electron transfer. However, owing to its centrosymmetric crystal structure, no macroscopic polarization is observed in this compound. Polar molecules with a [Co^2+^_HS_-dhbq^2−^-Co^3+^_LS_] structure align in an antiferroelectric way.

Meanwhile, macroscopic polarization changes stemming from intramolecular electron transfer between Co and dhbq are observed in the heterometallic [CrCo] dinuclear complex with chiral cth ligand [(Cr(*SS*-cth))(Co(*RR*-cth))(*μ*-dhbq)](PF_6_)_3_ (Fig. 1 and 12, *SS*-cth = *SS* isomer of cth, *RR*-cth = *RR* isomer of cth), which possesses a polar crystal structure (Fig. 12c).^87,88^ Note that homometallic dinuclear complexes such as [CoCo] complex have been reported before.^89,90^ The selective synthesis of the heterometallic [CrCo] complex was achieved by mixing a Cr complex with an enantiopure *SS*-cth ligand and a Co complex with an enantiopure *RR*-cth ligand. Furthermore, the left-handed chiral ligands preferentially interact with their right-handed counterparts, affording a polar crystal structure where the [CrCo] complex is aligned along the crystallographic *b*-direction as shown in Fig. 12c. The [CrCo] complex exhibits electron transfer at approximately 360 K (Fig. 12b), and the dipole moments of [Co^3+^_LS_-dhsq^3−^-Cr^3+^] at the low-temperature phase and [Co^2+^_HS_-dhbq^2−^-Cr^3+^] at the high-temperature phase are 0.06 and 9.07 Debye, respectively:[Co^3+^_LS_-dhsq^3−^-Cr^3+^] ⇄ [Co^2+^_HS_-dhbq^2−^-Cr^3+^]

The electronic states of the Co and Cr ions in the low-temperature phase are expressed as Co^3+^_LS_ (t_2g_^6^e_g_^0^, *S* = 0) and Cr^3+^ (t_2g_^3^e_g_^0^, *S* = 3/2), where Cr^3+^ (*S* = 3/2) interacts antiferromagnetically with the dhsq^3−^ radical (*S* = 1/2), representing the *S* = 1 ground state. The electronic states in the high-temperature phase are expressed as Co^2+^_HS_ (t_2g_^5^e_g_^2^, *S* = 3/2), Cr^3+^ (t_2g_^3^e_g_^0^, *S* = 3/2), and dhbq^2−^ (*S* = 0). Since the [CrCo] molecules are aligned in the same direction, intramolecular electron transfer leads to macroscopic polarization changes (Fig. 12c). Pyroelectric current is detected at around 360 K (Fig. 12b).^91^ The [CrCo] complex is not a ferroelectric material; however, it exhibits electron transfer-induced polarization change, representing the concept of electronic pyroelectrics. Since the dipole moment of the [CrCo] complexes at the low-temperature phase is approximately 0 Debye, polarization switching between on (*P* ≠ 0) and off (*P* = ∼0) states is realized as in ferroelectrics. The advantage of the electron transfer mechanism over the typical ion displacement and molecular reorientation mechanisms is that electron transfer can be directly manipulated *via* electronic excitation with light, resulting in an ultrafast polarization change. Femtosecond time-resolved absorption spectroscopy measurements revealed that the polarization change occurs at about 280 fs after photoexcitation.^92^ Furthermore, the advantage of pyroelectrics (nonferroelectrics) over ferroelectrics is that a domain with different polarization directions is not formed, meaning that all molecules undergo directional electron transfer (vectorial electron transfer) even in the absence of an electric field. The photo-induced polarization switching would be used as the mechanism for ultrafast photo-control of even-order nonlinear optical effects such as second harmonic generation.

The analogous complex [(Co(*RR*-cth))(Ga(*SS*-cth))(dhbq)](PF_6_)_3_ also exhibits polarization change *via* electron transfer; [Co^3+^_LS_-dhsq^3−^-Ga^3+^] ⇄ [Co^2+^_HS_-dhbq^2−^-Ga^3+^].^91^ Pyroelectric measurements reveal that large pyroelectric effect is detected at approximately 222 K, at which vectorial electron transfer occurs in the crystal. The polarization change (Δ*P*) *via* the electron transfer is about 2.9 μC cm^−2^, which is comparable to that of typical ferroelectrics such as triglycine sulfate (TGS, Δ*P* = 3.8 μC cm^−2^) being used as IR detector. Furthermore, the [CoGa] compounds exhibit photoinduced electron transfer and resultant electron transfer state is trapped as a metastable state at low temperature, where the lifetime of the metastable state is approximately 3.5 × 10^4^ s at 10 K. This means that the [CoGa] compounds have the optical memory property of electric polarization. Electric current is generated during the relaxation process from the metastable state [Co^2+^_HS_-dhbq^2−^-Ca^3+^] to ground state [Co^3+^_LS_-dhsq^3−^-Ca^3+^]. This means photoenergy is converted to electrical energy *via* photoinduced polarization change, which is distinct from typical pyroelectric compounds that exhibit the conversion of thermal energy into electricity.

The complex [(Fe(*RR*-cth))(Co(*SS*-cth))(dhbq)](X)_3_ (X = PF_6_ or AsF_6_) also exhibits polarization change *via* successive spin transition and electron transfer processes. The polarization change during spin transition and electron transfer is detected as pyroelectric current.^25,93,94^ The [FeCo] complex exhibits magnetoelectric effect.^95^ Electric polarization switching *via* spin transition induced by pulsed magnetic field is observed. Polarization change *via* electron transfer in response to temperature and light has been also observed in a mononuclear Co valence tautomeric complex, [Co(phendiox)(*rac*-cth)](ClO_4_)·0.5EtOH (H_2_ phendiox = 9,10-dihydroxyphenanthrene, *rac*-cth = racemic cth).^25,96^

## 5. Summary and outlook

In this article, representative examples of electronic ferroelectrics and electronic pyroelectrics have been introduced. Electronic polarization switching *via* charge ordering, neutral-to-ionic transition, and metal-to-metal electron transfer is observed in electronic ferroelectrics. However, only a limited number of electronic ferroelectrics, including oxides, have been reported so far. Therefore, electronic ferroelectrics with superior polarization properties should be synthesized. In particular, the development of room temperature electronic ferroelectrics with enhanced durability is essential to realize new applications. To this aim, a design strategy to develop electronic ferroelectrics, especially of coordination compounds, should be firmly established. As a result, electronic ferroelectrics with comparable or better properties than those of typical ferroelectrics functioning *via* ion displacement or molecular reorientation mechanisms could be synthesized.

Furthermore, electronic pyroelectrics are promising materials in the development of novel polarization switching compounds. The combination of the synthesis of molecules that exhibit electron transfer and the control of the electron transfer direction in crystals has enabled the development of pyroelectrics showing polarization switching *via* vectorial electron transfer. Since this is a bottom-up approach, it could lead to a variety of polarization-switchable materials based on the molecular design. Larger changes in macroscopic polarization than those reported to date can be expected using molecules that exhibit substantial changes in the molecular dipole moment. Recently, numerous photoswitchable compounds in electric polarization,^97–102^ including molecular systems,^103–106^ have been reported for application in memory devices. When molecular units exhibit photoinduced redox isomerization *via* electron transfer at room temperature, the electronic pyroelectrics exhibit optical memory property in electronic polarization. Realizing long-life time of the photoinduced metastable state *via* electron transfer at room temperature is a challenge. Although several promising approaches have been made along this line in the field of molecular photomagnetism and artificial photosynthetic systems,^107,108^ much further improvements are required for practical application. Meanwhile, when the molecular units have magnetic field effects, the electronic pyroelectrics exhibit magnetoelectric properties. Although polarization change *via* spin transition induced by a magnetic field has been reported,^109–113^ to our knowledge, the major contribution to the polarization change is a molecular structural change. Coupling the electron transfer (electron redistribution) with spin transition would lead to a large change in electronic polarization, which would be the major contributor to the polarization change, rather than ionic polarization or conformational polarization. Furthermore, various temperature-dependent polarization properties can be realized, including thermally induced polarization inversion. The development of compounds exhibiting photoinduced and magnetic field-induced polarization inversion is also expected. The concept of electronic ferroelectrics/pyroelectrics can be extended to electronic piezoelectrics, in which the mechanism of the piezoelectric effect and the inverse piezoelectric effect is electron transfer instead of ion displacement and molecular reorientation. New functionalities can be expected to emerge from electronic polarization based on the electron degrees of freedom.

## Author contributions

All authors equally contributed to the manuscript preparation and writing.

## Conflicts of interest

There are no conflicts to declare.

## Supplementary Material

## References

[cit1] Yao Z. S., Tang Z., Tao J. (2020). Bistable molecular materials with dynamic structures. Chem. Commun..

[cit2] Coronado E., Espallargas G. M. (2013). Dynamic magnetic MOFs. Chem. Soc. Rev..

[cit3] Akutagawa T., Takeda T., Hoshino N. (2021). Dynamics of proton, ion, molecule, and crystal lattice in functional molecular assemblies. Chem. Commun..

[cit4] Sato O. (2016). Dynamic molecular crystals with switchable physical properties. Nat. Chem..

[cit5] Naumov P. (2020). *et al.*, The Rise of the Dynamic Crystals. J. Am. Chem. Soc..

[cit6] Gransbury G. K. and Boskovic C., Valence Tautomerism in d-block complexes, Encyclopedia of Inorganic and Bioinorganic Chemistry, 2021, Wiley, 10.1002/9781119951438.eibc2785

[cit7] Huang W., Ma X., Sato O., Wu D. (2021). Controlling dynamic magnetic properties of coordination clusters: via switchable electronic configuration. Chem. Soc. Rev..

[cit8] Horiuchi S., Ishibashi S. (2020). Hydrogen-Bonded Small-Molecular Crystals Yielding Strong Ferroelectric and Antiferroelectric Polarizations. J. Phys. Soc. Jpn..

[cit9] Miyasaka H. (2021). Charge Manipulation in Metal-Organic Frameworks: Toward Designer Functional Molecular Materials. Bull. Chem. Soc. Jpn..

[cit10] Meng Y. S., Sato O., Liu T. (2018). Manipulating Metal-to-Metal Charge Transfer for Materials with Switchable Functionality. Angew. Chem., Int. Ed..

[cit11] Dey B., Chandrasekhar V. (2022). Fe^II^ spin crossover complexes containing N_4_O_2_ donor ligands. Dalton Trans..

[cit12] Harada J. (2021). Plastic/ferroelectric molecular crystals: ferroelectric performance in bulk polycrystalline forms. APL Mater..

[cit13] Han X. B., Chai C. Y., Liang B. D., Fan C. C., Zhang W. (2022). Ferroic phase transition molecular crystals. Crystengcomm.

[cit14] Wei X. K. (2022). *et al.*, Progress on Emerging Ferroelectric Materials for Energy Harvesting, Storage and Conversion. Adv. Energy Mater..

[cit15] Zhao L. (2021). *et al.*, Switching the magnetic hysteresis of an [Fe^II^–NC–W^v^]-based coordination polymer by photoinduced reversible spin crossover. Nat. Chem..

[cit16] Liu H. Y. (2022). *et al.*, Ferroelectricity in organic materials: from materials characteristics to de novo design. J. Mater. Chem. C.

[cit17] Park C., Lee K., Koo M., Park C. (2021). Soft Ferroelectrics Enabling High-Performance Intelligent Photo Electronics. Adv. Mater..

[cit18] Bergenti I. (2022). Recent advances in molecular ferroelectrics. J. Phys. D: Appl. Phys..

[cit19] Hu Y., Ren S. (2020). Electroresistance and electro-optic effects in molecular ferroelectrics. APL Mater..

[cit20] Ishihara S. (2014). Electronic ferroelectricity in molecular organic crystals. J. Phys.: Condens.Matter.

[cit21] Tomić S., Dressel M. (2015). Ferroelectricity in molecular solids: a review of electrodynamic properties. Rep. Prog. Phys..

[cit22] Lunkenheimer P., Loidl A. (2015). Dielectric spectroscopy on organic charge-transfer salts. J. Phys.: Condens.Matter.

[cit23] Kirova N., Brazovskii S. (2016). Electronic ferroelectricity in carbon based materials. Synth. Met..

[cit24] Akiyoshi R., Hayami S. (2022). Ferroelectric coordination metal complexes based on structural and electron dynamics. Chem. Commun..

[cit25] Paul A., Konar S. (2022). Electronic pyroelectricity: the interplay of valence tautomerism and spin transition. J. Mater. Chem. C.

[cit26] Zhang W., Xiong R. G. (2012). Ferroelectric Metal-Organic Frameworks. Chem. Rev..

[cit27] Takahashi T., Nogami Y., Yakushi K. (2006). Charge ordering in organic conductors. J. Phys. Soc. Jpn..

[cit28] De Souza M., Pouget J. P. (2013). Charge-ordering transition in (TMTTF)_2_X explored via dilatometry. J. Phys.: Condens.Matter.

[cit29] Monceau P., Nad F. Y., Brazovskii S. (2001). Ferroelectric mott-hubbard phase of organic (TMTTF)_2_X conductors. Phys. Rev. Lett..

[cit30] Nad F., Monceau P. (2006). Dielectric response of the charge ordered state in quasi-one-dimensional organic conductors. J. Phys. Soc. Jpn..

[cit31] Rösslhuber R. (2018). *et al.*, Structural and electronic properties of (TMTTF)_2_X salts with tetrahedral anions. Crystals.

[cit32] Chow D. S. (2000). *et al.*, Charge ordering in the TMTTF family of molecular conductors. Phys. Rev. Lett..

[cit33] Köhler B., Rose E., Dumm M., Untereiner G., Dressel M. (2011). Comprehensive transport study of anisotropy and ordering phenomena in quasi-one-dimensional (TMTTF)_2_X salts (X=PF_6_, AsF_6_, SbF_6_, BF_4_, ClO_4_, ReO_4_). Phys. Rev. B: Condens. Matter Mater. Phys..

[cit34] Dressel M., Dumm M., Knoblauch T., Masino M. (2012). Comprehensive Optical Investigations of Charge Order in Organic Chain Compounds (TMTTF)_2_X. Crystals.

[cit35] Kitou S. (2017). *et al.*, Successive Dimensional Transition in (TMTTF)_2_PF_6_ Revealed by Synchrotron X-ray Diffraction. Phys. Rev. Lett..

[cit36] Itoh H. (2021). *et al.*, Charge correlations and their photoinduced dynamics in charge-ordered organic ferroelectrics. Phys. Rev. Res..

[cit37] Yu W. (2004). *et al.*, Electron-lattice coupling and broken symmetries of the molecular salt (TMTTF)_2_SbF_6_. Phys. Rev. B: Condens. Matter Mater. Phys..

[cit38] Giovannetti G., Nourafkan R., Kotliar G., Capone M. (2015). Correlation-driven electronic multiferroicity in TMTTF_2_-X organic crystals. Phys. Rev. B: Condens. Matter Mater. Phys..

[cit39] Pustogow A., Peterseim T., Kolatschek S., Engel L., Dressel M. (2016). Electronic correlations versus lattice interactions: Interplay of charge and anion orders in (TMTTF)_2_X. Phys. Rev. B.

[cit40] Bender K. (1984). *et al.*, Bis(ethylenedithiolo)tetrathiofulvalene. Mol. Cryst. Liq. Cryst..

[cit41] Dressel M., Tomić S. (2020). Molecular quantum materials: electronic phases and charge dynamics in two-dimensional organic solids. Adv. Phys..

[cit42] Kouda N., Eguchi K., Okazaki R., Tamura M. (2022). Anomalous scaling law for thermoelectric transport of two-dimension-confined electrons in an organic molecular system. Phys. Rev. Res..

[cit43] Kakiuchi T., Wakabayashi Y., Sawa H., Takahashi T., Nakamura T. (2007). Charge ordering in α-(BEDT-TTF)_2_I_3_ by synchrotron x-ray diffraction. J. Phys. Soc. Jpn..

[cit44] Yamamoto K. (2008). *et al.*, Strong optical nonlinearity and its ultrafast response associated with electron ferroelectricity in an organic conductor. J. Phys. Soc. Jpn..

[cit45] Yamamoto K., Kowalska A. A., Yakushi K. (2010). Direct observation of ferroelectric domains created by Wigner crystallization of electrons in α- [bis(ethylenedithio)tetrathiafulvalene]_2_I_3_. Appl. Phys. Lett..

[cit46] Lunkenheimer P. (2015). *et al.*, Ferroelectric properties of charge-ordered α-(BEDT-TTF)_2_I_3_. Phys. Rev. B: Condens. Matter Mater. Phys..

[cit47] Lunkenheimer P. (2012). *et al.*, Multiferroicity in an organic charge-transfer salt that is suggestive of electric-dipole-driven magnetism. Nat. Mater..

[cit48] Sedlmeier K. (2012). *et al.*, Absence of charge order in the dimerized kappa-phase BEDT-TTF salts. Phys. Rev. B: Condens. Matter Mater. Phys..

[cit49] Yamakawa H. (2021). *et al.*, Terahertz-field-induced polar charge order in electronic-type dielectrics. Nat. Commun..

[cit50] Kobayashi K. (2012). *et al.*, Electronic Ferroelectricity in a Molecular Crystal with Large Polarization Directing Antiparallel to Ionic Displacement. Phys. Rev. Lett..

[cit51] Horiuchi S., Kobayashi K., Kumai R., Ishibashi S. (2014). Ionic versus Electronic Ferroelectricity in Donor-Acceptor Molecular Sequences. Chem. Lett..

[cit52] Horiuchi S. , Ishibashi S. and Tokura Y., in Organic Ferroelectric Materials and Applications, ed. K. Asadi, Elsevier, 2022, ch. 2, pp. 7–46

[cit53] Torrance J. B., Vazquez J. E., Mayerle J. J., Lee V. Y. (1981). Discovery of a Neutral-to-Ionic Phase Transition in Organic Materials. Phys. Rev. Lett..

[cit54] Buron-Le Cointe M., Collet E., Toudic B., Czarnecki P., Cailleau H. (2017). Back to the structural and dynamical properties of neutral-ionic phase transitions. Crystals.

[cit55] Dressel M., Peterseim T. (2017). Infrared investigations of the neutral-ionic phase transition in TTF-CA and its dynamics. Crystals.

[cit56] Masino M., Castagnetti N., Girlando A. (2017). Phenomenology of the neutral-ionic valence instability in mixed stack charge-transfer crystals. Crystals.

[cit57] Morimoto T. (2021). *et al.*, Ionic to neutral conversion induced by resonant excitation of molecular vibrations coupled to intermolecular charge transfer. Phys. Rev. Res..

[cit58] Sunami K., Takehara R., Miyagawa K., Okamoto H., Kanoda K. (2022). Topological Excitations in Neutral–Ionic Transition Systems. Symmetry.

[cit59] Giovannetti G., Kumar S., Stroppa A., Van Den Brink J., Picozzi S. (2009). Multiferroicity in TTF-CA organic molecular crystals predicted through Ab initio calculations. Phys. Rev. Lett..

[cit60] Ishibashi S., Terakura K. (2010). First-principles study of spontaneous polarization in tetrathiafulvalene-p-chloranil (TTF-CA). Phys. B.

[cit61] Girlando A., Pecile C., Torrance J. B. (1985). A Key To Understanding Ionic Mixed Stacked Organic-Solids – Tetrathiafulvalene-Bromanil (TTF-BA). Solid State Commun..

[cit62] Kagawa F., Horiuchi S., Tokunaga M., Fujioka J., Tokura Y. (2010). Ferroelectricity in a one-dimensional organic quantum magnet. Nat. Phys..

[cit63] Liu X. L. (2021). *et al.*, Room-Temperature Magnetoelectric Coupling in Electronic Ferroelectric Film based on (n-C_3_H_7_)_4_N [Fe^III^Fe^II^(dto)_3_](dto = C_2_O_2_S_2_). J. Am. Chem. Soc..

[cit64] Kojima N. and Okazawa A., Molecular Magnetism of Metal Complexes and Light-Induced Phase Transitions, Modern Mössbauer Spectroscopy: New Challenges Based on Cutting-Edge Techniques, 2021, pp. 267–317, 10.1007/978-981-15-9422-9_6

[cit65] Itoi M. (2006). *et al.*, Charge-transfer phase transition and ferromagnetism of iron mixed-valence complexes (n-C_n_H_2n+1_)_4_N[Fe^II^Fe^III^(dto)_3_] (n = 3-6; dto = C_2_O_2_S_2_). Eur. J. Inorg. Chem..

[cit66] Ida H. (2012). *et al.*, Effect of nonmagnetic substitution on the magnetic properties and charge-transfer phase transition of an iron mixed-valence complex, (n-C_3_H_7_)_4_N[Fe^II^Fe^III^(dto)_3_] (dto = C_2_O_2_S_2_). Inorg. Chem..

[cit67] Enomoto M., Ida H., Okazawa A., Kojima N. (2018). Effect of transition metal substitution on the charge-transfer phase transition and ferromagnetism of dithiooxalato-bridged hetero metal complexes, (n-C_3_H_7_)_4_N[Fe^II^_1-x_Mn^II^_x_Fe^III^(dto)_3_]. Crystals.

[cit68] Nomura K., Kanetomo T., Enomoto M. (2022). Pressure-Induced Structural and Charge-Transfer Phase Transitions for a Two-Dimensional Mixed-Valence Iron(II,III) Coordination Polymer. Cryst. Growth Des..

[cit69] Nakamoto T. (2001). *et al.*, Heat capacity of the mixed-valence complex {[(n-C_3_H_7_)_4_N][Fe^II^Fe^III^(dto)_3_]}_∞_, phase transition because of electron transfer, and a change in spin-state of the whole system. Angew. Chem., Int. Ed..

[cit70] Kojima N., Enomoto M., Kida N., Kagesawa K. (2010). Progress of Multi Functional Properties of Organic-Inorganic Hybrid System, A (Fe^II^Fe^III^X_3_) (A = (n-C_n_H_2n+1_)_4_N, Spiropyran; X = C_2_O_2_S_2_, C_2_OS_3_, C_2_O_3_S). Materials.

[cit71] Sato O., Tao J., Zhang Y. Z. (2007). Control of Magnetic Properties through External Stimuli. Angew. Chem., Int. Ed..

[cit72] Monni N., Angotzi M. S., Oggianu M., Sahadevan S. A., Mercuri M. L. (2022). Redox-active benzoquinones as challenging ": non-innocent " linkers to construct 2D frameworks and nanostructures with tunable physical properties. J. Mater. Chem. C.

[cit73] You M. (2022). *et al.*, Thermally Induced Reversible Metal-to-Metal Charge Transfer in Mixed-Valence {Fe^III^_4_Fe^II^_4_} Cubes. CCS Chem..

[cit74] Cammarata M. (2021). *et al.*, Charge transfer driven by ultrafast spin transition in a CoFe Prussian blue analogue. Nat. Chem..

[cit75] Zhang J., Kosaka W., Kitagawa Y., Miyasaka H. (2021). A metal–organic framework that exhibits CO_2_-induced transitions between paramagnetism and ferrimagnetism. Nat. Chem..

[cit76] Yadav J., Mondal D. J., Konar S. (2021). High-temperature electron transfer coupled spin transition (ETCST) with hysteresis in a discrete [Fe_2_Co_2_] Prussian blue analogue. Chem. Commun..

[cit77] Stefanczyk O., Ohkoshi S. (2021). Photoswitchable high-dimensional Co^II^W^V^(CN)_8_ networks: Past, present, and future. J. Appl. Phys..

[cit78] Klokishner S., Ostrovsky S. (2021). Modeling of electron transfer phenomenon in the dinuclear {Fe(μ-CN)Co} complexes. J. Appl. Phys..

[cit79] Gransbury G. K. (2020). *et al.*, Understanding the Origin of One- or Two-Step Valence Tautomeric Transitions in Bis(dioxolene)-Bridged Dinuclear Cobalt Complexes. J. Am. Chem. Soc..

[cit80] Meng Y. S., Liu T. (2019). Manipulating Spin Transition To Achieve Switchable Multifunctions. Acc. Chem. Res..

[cit81] Degayner J. A., Wang K., Harris T. D. (2018). A Ferric Semiquinoid Single-Chain Magnet via Thermally-Switchable Metal-Ligand Electron Transfer. J. Am. Chem.
Soc..

[cit82] Sato O., Iyoda T., Fujishima A., Hashimoto K. (1996). Photoinduced Magnetization of a Cobalt Iron Cyanide. Science.

[cit83] Sato O. (2001). *et al.*, Photo-Induced Long-Lived Intramolecular Electron Transfer in a Co Valence Tautomeric Complex. Chem. Lett..

[cit84] Sato O., Cui A. L., Matsuda R., Tao J., Hayami S. (2007). Photo-induced Valence Tautomerism in Co Complexes. Acc. Chem. Res..

[cit85] Li G. L. (2016). *et al.*, Influence of Intermolecular Interactions on Valence Tautomeric Behaviors in Two Polymorphic Dinuclear Cobalt Complexes. Chem.–Eur. J..

[cit86] Chegerev M. G., Starikova A. A. (2021). Electronic Lability of Quinonoid-Bridged Dinuclear 3d-Metal Complexes with Tetradentate N-Donor Bases. Eur. J. Inorg. Chem..

[cit87] Kanegawa S. (2016). *et al.*, Directional Electron Transfer in Crystals of CrCo Dinuclear Complexes Achieved by Chirality-Assisted Preparative Method. J. Am. Chem. Soc..

[cit88] Klokishner S., Reu O., Roman M. (2021). Valence tautomeric transformation in the [CrCo] compound: exploration of cooperative interactions. Phys. Chem. Chem. Phys..

[cit89] Carbonera C., Dei A., Letard J. F., Sangregorio C., Sorace L. (2004). Thermally and Light-Induced Valence Tautomeric Transition in a Dinuclear Cobalt-Tetraoxolene Complex. Angew. Chem., Int. Ed..

[cit90] Dei A., Gatteschi D., Pardi L., Russo U. (1991). Tetraoxolene Radical Stabilization by the Interaction with Transition-Metal Ions. Inorg. Chem..

[cit91] Sadhukhan P. (2023). *et al.*, Energy conversion and storage via photoinduced polarization change in non-ferroelectric molecular [CoGa] crystals. Nat. Commun..

[cit92] Kuramochi H. (2020). *et al.*, Femtosecond Polarization Switching in the Crystal of a CrCo Dinuclear Complex. Angew. Chem., Int. Ed..

[cit93] Sadhukhan P. (2021). *et al.*, Manipulating electron redistribution to achieve electronic pyroelectricity in molecular FeCo crystals. Nat. Commun..

[cit94] Yamamoto K., Kawasaki A., Chinen T., Ryugo K. (2021). Temperature-modulated pyroelectricity measurements of a thin ferroelectric crystal with in-plane polarization and the thermal analysis based on one-dimensional layer models. Crystals.

[cit95] Zhang X. (2023). *et al.*, Magnetoelectricity Enhanced by Electron Redistribution in a Spin Crossover [FeCo] Complex. J. Am. Chem. Soc..

[cit96] Wu S. Q. (2020). *et al.*, Macroscopic Polarization Change via Electron Transfer in a Valence Tautomeric Cobalt Complex. Nat. Commun..

[cit97] Nova T. F., Disa A. S., Fechner M., Cavalleri A. (2019). Metastable ferroelectricity in optically strained SrTiO_3_. Science.

[cit98] Liou Y. D. (2019). *et al.*, Deterministic optical control of room temperature multiferroicity in BiFeO_3_ thin films. Nat. Mater..

[cit99] Rubio-Marcos F. (2018). *et al.*, Reversible optical control of macroscopic polarization in ferroelectrics. Nat. Photonics.

[cit100] Li T. (2018). *et al.*, Optical control of polarization in ferroelectric heterostructures. Nat. Commun..

[cit101] Yang M. M., Alexe M. (2018). Light-Induced Reversible Control of Ferroelectric Polarization in BiFeO_3_. Adv. Mater..

[cit102] Kreisel J., Alexe M., Thomas P. A. (2012). A photoferroelectric material is more than the sum of its parts. Nat. Mater..

[cit103] Tang Y. Y., Zeng Y. L., Xiong R. G. (2022). Contactless Manipulation of Write-Read-Erase Data Storage in Diarylethene Ferroelectric Crystals. J. Am. Chem. Soc..

[cit104] Peng H., Qi J. C., Liao W. Q. (2022). Optically Controlled Polarization Switching in an Organic Ferroelectric with Light- and Temperature-Triggered Phase Transitions. Chem. Mater..

[cit105] Tang Y. Y. (2021). *et al.*, Optical Control of Polarization Switching in a Single-Component Organic Ferroelectric Crystal. J. Am. Chem. Soc..

[cit106] Su S. Q. (2022). *et al.*, Photoinduced Persistent Polarization Change in a Spin Transition Crystal. Angew. Chem., Int. Ed..

[cit107] Cai L. Z. (2015). *et al.*, Photochromism and Photomagnetism of a 3d-4f Hexacyanoferrate at Room Temperature. J. Am. Chem. Soc..

[cit108] Fukuzumi S., Ohkubo K., Suenobu T. (2014). Long-lived charge separation and applications in artificial photosynthesis. Acc. Chem. Res..

[cit109] Owczarek M. (2022). *et al.*, Near-Room-Temperature Magnetoelectric Coupling via Spin Crossover in an Iron(II) Complex. Angew. Chem., Int. Ed..

[cit110] Jakobsen V. B. (2022). *et al.*, Domain Wall Dynamics in a Ferroelastic Spin Crossover Complex with Giant Magnetoelectric Coupling. J. Am. Chem. Soc..

[cit111] Jakobsen V. B. (2021). *et al.*, Giant Magnetoelectric Coupling and Magnetic-Field-Induced Permanent Switching in a Spin Crossover Mn(III) Complex. Inorg. Chem..

[cit112] Chikara S. (2019). *et al.*, Magnetoelectric behavior via a spin state transition. Nat. Commun..

[cit113] Otsuki Y., Kimura S., Awaji S., Nakano M. (2019). Magnetocapacitance effect and magnetostriction by the field-induced spin-crossover in [Mn^III^(taa)]. AIP Adv..

